# Associations between Mental Resilience, Mood, Coping, Personality, and Hangover Severity

**DOI:** 10.3390/jcm11082240

**Published:** 2022-04-17

**Authors:** Chantal Terpstra, Joris C Verster, Andrew Scholey, Sarah Benson

**Affiliations:** 1 Centre for Human Psychopharmacology, Swinburne University, Melbourne, VIC 3122, Australia; andrew@scholeylab.com (A.S.); sarahbenson@swin.edu.au (S.B.); 2 Division of Pharmacology, Utrecht Institute for Pharmaceutical Sciences (UIPS), Utrecht University, 3584 CG Utrecht, The Netherlands; j.c.verster@uu.nl

**Keywords:** alcohol, hangover, mental resilience, mood, coping, personality

## Abstract

Extensive research exists on relationships between psychological constructs and alcohol consumption. However, research on relationships with hangover severity remains limited. This study aimed to assess the associations between mental resilience, mood (i.e., depression, anxiety, and stress), coping, personality, and hangover severity. A total of N = 690 participants completed an online survey by answering questions regarding their demographics, alcohol use, hangover prevalence and severity, and several psychological assessments (Brief Resilience Scale, DASS-21, Brief Cope, and Brief Version of the Big Five Personality Inventory). Significant associations were found between hangover severity and mental resilience, mood, and avoidant coping. Higher levels of mental resilience were associated with less severe hangovers, whereas poorer mood was associated with more severe hangovers. No significant associations were found with personality traits. These findings demonstrate that several associations between psychological constructs and hangover severity exist and suggest a role of psychological factors in the pathology of the alcohol hangover. As our findings contrast with the results of previous studies that did not report an association between mental resilience and the presence and severity of hangovers, further research is warranted.

## 1. Introduction

Excessive alcohol use is closely related to poor mental health [1] and can result in the development of psychological distress, mental disorders, and suicide risk [2,3,4]. Conversely, alcohol consumption can also function as a coping mechanism to manage stressful situations, depression, or anxiety [5,6] and provide a temporary relief [1]. Alcohol consumption and mental health, thus, constitute a bidirectional association.

Individuals with hazardous alcohol consumption are more likely to report psychological distress than those with lower alcohol intake [7]. Although depression and anxiety are both associated with alcohol use, associations may be more robust for depression symptoms [8,9]. Both clinical and population-based studies demonstrate that heavy drinking relates to higher depression levels [10]. Anxiety and stress have been found to causally relate to both alcohol craving and consumption [11,12,13]. Stress is associated with increased alcohol use/heavy drinking, with a positive association between the number of stressors and alcohol intake [14]. Stress also relates to poorer mental health [15], and, therefore, the use of negative coping styles, such as increased alcohol consumption, are more likely to develop [16].

Coping involves using thoughts and behaviors to manage internal and external stressful situations [17] or approaches to manage exposure to stressors [18]. Adaptive (problem-focused/active) coping styles predict advantageous mental health outcomes and less alcohol use, whereas maladaptive (avoidant/passive) coping styles are associated with increased alcohol use [19,20,21]. Additionally, stressful situations are possibly more easily managed by individuals with high levels of mental resilience. Mental resilience refers to the ability to recover or bounce back from stressful events [22,23]. High levels of mental resilience are associated with lower stress levels and lower hazardous drinking levels [24].

Furthermore, mental resilience mediates the relationship between stress and binge drinking [25]. Low resilience levels may, thus, result in increased ineffective coping skills, such as increased alcohol use, to manage stressors [26]. Accordingly, negative associations exist for alcohol use and resilience levels [27], and the ability to describe negative emotions (a mental resilience characteristic) is associated with decreases in alcohol consumption [28].

More intrinsic factors, such as personality, have also been associated with alcohol use. Neuroticism (high scorers tend to be anxious, depressed, angry, and insecure [29]) demonstrated strong correlations with coping-motivated drinking, whereas extraversion (high scorers tend to be sociable, talkative, assertive, and active [29]) shows stronger correlations with social reasons to drink [30,31]. Extraversion correlates with alcohol consumption, whereas neuroticism correlates with negative drinking-related consequences [32]. Some research suggested that neuroticism is associated with hazardous drinking to minimize emotions [33]. Low levels of conscientiousness (high scorers tend to be careful, thorough, responsible, organized, and honest [29]) and agreeableness (high scorers tend to be good-natured, compliant, modest, gentle, and cooperative [29]) are linked with increased hazardous drinking [34].

Even though extensive research, thus, exists on relationships between alcohol consumption and psychological factors, research assessing these relationships with alcohol hangovers is still extremely limited [35]. The alcohol hangover is the most frequently reported adverse event of alcohol consumption [36] and is defined as the combination of negative mental and physical symptoms, which can be experienced after a single episode of alcohol consumption, starting when blood alcohol concentration (BAC) approaches zero [37,38]. Alcohol hangovers come with substantial consequences for the economy, health, and society [39]. Furthermore, alcohol hangovers increase accident risks due to impairments in psychomotor and cognitive performance [40], which could impact daily activities, such as driving a car and riding a bicycle [41,42,43].

A recent regression analysis showed that depression symptoms are associated with high current and future vulnerability to hangover symptoms after drinking [44], and greater self-reported hangover severity positively correlates with depression [45]. Alcohol hangovers are associated with increased anxiety as well [46], and a recent survey found positive correlations between anxiety levels on the hangover day and scores on the Alcohol Use Disorders Identification Test (AUDIT) among highly shy individuals [47]. However, recent research demonstrated that, even though anxiety and depression symptoms can be present in the hangover phase, both presence and severity are generally low, and other symptoms (i.e., headache, fatigue, etc.) are more frequently reported and more severe [48]. Feeling stressed while experiencing a hangover is also related to increased hangover severity [35]. Associations between mental resilience and hangover presence and severity were not significant in previous research [49,50]. Research on coping styles and hangover severity is scarce. However, a recent study linked higher levels of pain catastrophizing, i.e., the tendency to ruminate and overestimate pain experiences, to experiencing more severe hangovers [51].

Very limited research exists on hangover severity and personality traits, and most research was conducted decades ago. High levels of neuroticism predicted hangovers [52], and a significant association was found between personality and problem drinking for individuals experiencing many hangover symptoms, but not for those experiencing few symptoms [53]. However, it is essential to note that several limitations in this study complicate the interpretation of the presented findings [35,52].

Firstly, participants who reported not being drunk (yet could still have had a hangover) were excluded from the analyses. Secondly, 23% of participants experienced no hangover symptoms but remained in the sample, which may have affected the results [35]. Finally, the hangover scale used in this study contained items that are intoxication-related but not related to hangovers (i.e., blackouts) and omitted several core symptoms of the hangover state (e.g., fatigue/nausea), while including symptoms that are irrelevant to hangovers (e.g., thoughts of suicide) [35,54]. A recent study concluded that such composite hangover scales are less accurate than single item assessments of overall hangover severity [55].

Limited research assesses psychological factors and hangover severity, and more studies need to be conducted to elucidate to what extent psychological factors and personality impact hangover severity [35,56]. Therefore, this study aimed to investigate whether mental resilience, mood (i.e., depression, anxiety, stress), coping, and personality are associated with hangover severity among an Australian population.

## 2. Materials and Methods

### 2.1. Method

The study was approved by the Swinburne Human Research Ethics Committee (Reference 20202783-5699, approval date 14 December 2020) and was conducted in accordance with the Declaration of Helsinki. Informed consent was obtained from all participants.

### 2.2. Design

This online survey was conducted among an Australian population and assessed associations between several psychological constructs (mental resilience, mood, coping, and personality) and hangover severity. The survey was conducted from 27 January until 7 February 2021.

### 2.3. Participants

N = 690 participants completed the survey. Participants who were not residing in Australia (N = 4), withdrew consent (N = 6), used illicit drugs while consuming alcohol (N = 31), and provided unreliable data (N = 1) were removed. The final sample consisted of 648 participants (25% male, 73.6% female, and 1.4% referred to themselves as other). The majority of participants lived in Victoria (63.4%) and generally had tertiary (53.6%) or postgraduate (29.0%) education levels. The majority of participants reported Australian (65.0%) and European ethnicity (19.2%).

### 2.4. Measures

#### 2.4.1. Demographics

Participants answered questions regarding demographics (gender, age, ethnicity, and highest education) and illicit drugs.

#### 2.4.2. Alcohol Consumption Questions

Participants were questioned on their alcohol intake in the past 30 days. Alcohol consumption was defined by using standardized Australian alcohol units (one standard drink = 10 g of pure alcohol). Illustrations of standard drinks were used to illustrate the sizes of standard drinks. The consumption questions assessed the frequency and quantity of alcohol consumed. Specifically, participants were asked the number of standard drinks and the number of hours spent drinking. 

#### 2.4.3. Hangover Prevalence/Severity

Hangover prevalence was assessed with a yes/no question: “Have you had a hangover in the past 30 days”, relating to the last drinking occasion. Hangover severity was assessed in the past 30 days with the Alcohol Hangover Severity Scale (AHSS), developed by Penning et al. [57]. The scale consists of 12 items (i.e., fatigue, apathy, concentration problems, clumsiness, confusion, thirst, sweating, shivering, stomach pain, nausea, dizziness, and heart pounding). Each item can be rated on a scale from 0 (absent) to 10 (extreme). Total hangover severity consists of the mean score across the 12 items [57]. Higher scores indicate higher hangover severity. The 12-item AHSS was found to be reliable (Cronbach’s α = 0.84), and predictive validity was high (92.4%) [57].

#### 2.4.4. Mental Resilience

Mental resilience was assessed with the Brief Resilience Scale (BRS) [58] to evaluate the perceived ability to recover from stress or the ability to bounce back. The scale consists of 6 items, and each item can be rated on a scale from 1 (“Strongly disagree”) to 5 (“Strongly agree”). The BRS is scored by recoding items 2, 4, and 6 and averaging the six items [58]. Higher scores indicate higher levels of mental resilience. The 6-item BRS was found to be reliable (Cronbach’s α = 0.80–0.91) [58]. The total score of the BRS was used for this study.

#### 2.4.5. Mood

The DASS-21 was used to assess depression, anxiety, and stress symptoms in general (in the past seven days) and is a widely used measure [59]. The scale consists of 21 questions, comprising 7 items on the three subscales (depression, anxiety, and stress). Each item can be rated on a scale from 0 (“Did not apply to me at all”) to 3 (“Applied to me very much or most of the time”). Sum scores for the subscales are calculated by adding scores on items per subscale and multiplying them by 2. The DASS-21 was found to be reliable on the depression scale (Cronbach’s Alpha = 0.82), the anxiety scale (Cronbach’s Alpha = 0.90), and the stress scale (Cronbach’s Alpha = 0.90) [60].

#### 2.4.6. Coping

Coping styles were assessed with the Brief Cope [61]. The scale assesses effective and ineffective ways to cope with stressful life events. The scale consists of 28 items, which can be rated on a scale from 1 (“I haven’t been doing this at all”) to 4 (“I have been doing this a lot”). The scale consists of 14 subscales, with three main coping styles (problem-focused, emotion-focused, and avoidant). Sum scores for the subscales are calculated by averaging the scores on the items [62]. The Brief Cope was found to be reliable (Cronbach’s Alpha = 0.50–0.90).

#### 2.4.7. Personality

Personality traits were assessed with the Big Five Inventory 10 items (BFI-10) [63]. This 10-item scale measures the Big Five personality traits: extraversion, agreeableness, conscientiousness, neuroticism, and openness. Each item can be rated on a scale from 1 (strongly disagree) to 5 (strongly agree). The BFI-10 is scored by recoding items 1, 3, 4, 5, and 7 and calculating an average of the two items per subscale. The 10-item BFI demonstrates acceptable reliability, with a test–retest correlations of r = 0.49–r = 0.79 [63].

### 2.5. Procedure

Participants were recruited via word of mouth and advertisements on social media. Participation was voluntary. Participants provided informed consent by agreeing to the survey terms online, after which they completed the online questionnaire. Upon completion, participants had the option to withdraw or submit responses and to choose to go into the draw to win 1 out of 5 $100 VISA prepaid gift cards by providing an email address.

### 2.6. Statistical Analysis

Data were collected by using SurveyMonkey and analyzed by using the Statistical Package for the Social Sciences version (26) (SPSS Inc., Chicago, IL, USA). Initially, the data were screened for participants who did not meet the criteria, i.e., residents outside Australia were excluded. Additionally, several participants used alcohol and illicit drugs simultaneously in the past 30 days and were also removed. The mean, standard deviation, and frequency distributions of alcohol consumption behaviors, hangover prevalence, and severity were calculated. To assess possible differences between the subset of alcohol consumers and alcohol abstainers, an independent samples *t*-test was conducted. Because of smaller sample sizes in the hangover analyses and several variables not following normal distribution, nonparametric partial correlations were used. Nonparametric partial correlations were computed between psychological variables and hangover severity, controlling for the greatest number of drinks. Results were considered significant if *p* < 0.05.

## 3. Results

### 3.1. Alcohol Consumption

A total of 75.4% reported alcohol consumption (N = 477), and 24.6% reported alcohol abstinence (N = 156). The data of those who consume alcohol were further evaluated. A general description of the participant demographics and typical drinking patterns is provided in Table 1.

### 3.2. Psychological Assessment

Several psychological constructs were assessed. An independent *t*-test was conducted to compare the scores on the psychological constructs for alcohol consumers and alcohol abstainers. A significant difference was found in scores on extraversion between alcohol consumers (M = 3.07, SD = 1.07) and alcohol abstainers (M = 2.78, SD = 1.07), *t* (577) = 2.74, *p* < 0.01. No other significant differences were found. The means and standard deviations of the psychological constructs in both alcohol consumers and alcohol abstainers are shown in Table 2.

### 3.3. Alcohol Hangover Prevalence and Severity

Hangover prevalence was assessed in the past 30 days, with 23.4% (N = 90) reporting a hangover. The mean and standard deviations of 12 individual hangover items and the overall hangover severity can be found in Table 3.

As can be seen in Table 4, significant partial correlations (controlling for greatest number of drinks) with Bonferroni corrections were found between mental resilience and hangover severity (r = −0.305, *p* = 0.004), indicating that, with increasing mental resilience, drinkers reported significantly less hangover severity. Significant partial correlations were also found between depression (r = 0.538, *p* < 0.001), anxiety, (r = 0.602, *p* < 0.001), and stress (r = 0.536, *p* < 0.001) and hangover severity, indicating that, with increasing levels of depression, anxiety, and stress, drinkers reported significantly more severe hangovers. Avoidant coping (r = 0.386, *p* < 0.001) was also positively correlated with hangover severity, indicating that, with increasing levels of avoidant coping, drinkers report significantly more severe hangovers. There were no significant partial correlations between hangover severity and problem-focused coping, emotion-focused coping, extraversion, agreeableness, conscientiousness, neuroticism, and openness to experiences.

## 4. Discussion

In contrast to previous findings [49,50], our data suggest that higher levels of mental resilience are associated with less hangover severity. A possible explanation for this association might relate to the association between higher levels of mental resilience and lower levels of stress and risky drinking behaviors [24]. The absence of risky drinking behaviors (e.g., binge drinking) could consequently result in lower hangover severity. An explanation for contrasting results with previous findings could be that one former study assessed differences in mental resilience in subgroups of hangover-sensitive and hangover-resistant drinkers [49], whereas the current study assessed mental resilience with hangover severity in the full sample of drinkers. Additionally, our study used the total score of the AHSS for hangover severity, while previous research used a one-item hangover severity score [50]. Future research should use a one-item hangover severity score to improve the accuracy of this assessment [55]. Furthermore, in the previous studies, the participants were all students, whereas the current study was conducted among the general Australian population (age range of 18–88) [49,50]. Previous research demonstrated that it is important to consider age when investigating the alcohol hangover, due to decreased frequency and severity in older individuals [64], as this may have differentially affected the current study as opposed to previous studies.

In contrast to previous findings [35], poorer mood (i.e., depression, anxiety, and stress) was associated with increased hangover severity. Future research should therefore further investigate the possible relationship between baseline (general) mood and the presence and severity of alcohol hangover. Despite this discrepancy, it is clear from previous research and ours that significant mood changes are evident during the hangover state.

Our findings indicate that drinkers with higher scores on avoidant coping report more severe hangovers. An explanation for this finding could be that individuals who use avoidant coping skills are likely to use alcohol as a coping mechanism to manage stressful situations, depression, or anxiety [5,6] and can function as a temporary relief [1]. Increased and risky drinking behaviors may consequently result in more negative consequences after drinking (i.e., alcohol hangover). Additionally, when individuals are in a hangover state and, thus, experience a combination of negative mental and physical symptoms [37,38], this negative state may make it increasingly difficult to implement adaptive coping strategies, such as positive reframing, acceptance, or seeking emotional support [61]. Positive reframing (e.g., feeling ill, but expressing gratitude for the night out), acceptance (e.g., feeling ill currently, but knowing it is temporary and will pass), and seeking emotional support (e.g., talking to a friend who is also currently hungover) could possibly positively impact the perceived hangover severity. More research is needed to further explore the implementation of different coping strategies and their influence on hangover severity.

Historical findings suggested that high levels of neuroticism predicted hangover [52] and found significant associations between personality and problem drinking for individuals experiencing many hangover symptoms, but not for those experiencing few symptoms [53]. As previously discussed, several limitations complicated the interpretation of these findings and may explain the absence of associations between personality traits and hangover severity in this study. Our findings are in line with assessments of neuroticism in more recent research [35].

It is important to note that mediating relationships are likely to exist between the psychological variables, and this could consequently impact the associations with hangover severity. For example, avoidant strategies are typically linked to anxiety and depressive symptoms [65,66]. Furthermore, personality type may also influence the choice of coping strategies indirectly (influences on the severity of stressors and effectiveness of coping) or directly (how individuals engage or disengage with threats and stressors) [67]. More neurotic individuals may respond to stressors with disengagement, whereas highly social extraverts may seek more supportive coping [67]. Additionally, a link between mental health and personality also exists. Personality traits such as extraversion, neuroticism, and conscientiousness are linked to depression [68], and high neuroticism and low extraversion are markers of risk for anxiety disorders [69]. The relationships between the psychological variables and subsequent relationships with alcohol hangovers should be assessed further.

There are several limitations of the current study that warrant attention. Firstly, due to the cross-sectional nature of the study, no causality can be claimed. Secondly, the data were self-reported. Even though mood and personality were analyzed via validated questionnaires, a formal diagnosis of psychiatric comorbidities (e.g., alcohol use disorder or psychiatric diseases) has not been made. In future research, it would be interesting to evaluate the relationship between mood, personality, and hangover severity in patients with a formal diagnosis of alcohol-use disorders or psychiatric diseases. Future studies should also assess the possible impact of tolerance or familial risk of alcohol use disorder, particularly given the associations between familial risk of alcohol-related problems and development of alcohol-use disorder (AUD) [70,71]. Studies could also investigate the possible impact of social, demographic, and health variables, such as educational status, income, and body mass index, on hangover severity. Furthermore, the simultaneous use of psychoactive substances and alcohol can intensify or minimize the prevalence and severity of alcohol hangovers [72], and future research should include the co-use of illicit drugs and other psychoactive substances that were not assessed in the current study.

Other limitations of the study include commonly reported limitations in survey research, such as recall bias and socially desirable answering [35]. Furthermore, women were overrepresented in this sample, and the number of people reporting hangover severity was low. Future research should therefore aim to include diverse populations and larger sample sizes.

In conclusion, in the current study, higher levels of mental resilience were associated with experiencing less severe hangovers, whereas poorer mood was associated with experiencing more severe hangovers. No significant associations were found with personality traits. As these findings contrast those of previous research and suggest a role of psychological factors in the pathology of the alcohol hangover, further research is warranted.

## Figures and Tables

**Table 1:** Means and standard deviations for participant demographic and drinking characteristics.

	M (SD)
Age in years (range 18–88)	47.8 (18.2)
Days used alcohol (30 days)	11.3 (8.9)
Days drunk (30 days)	2.4 (3.4)
Days binged (30 days)	3.7 (5.3)
Greatest number of drinks (30 days)	6.0 (4.3)
Consumption duration (hours)	4.8 (2.6)
Overall hangover severity	3.8 (2.2)

**Table 2:** Means and standard deviations on psychological assessments.

Psychological Constructs	Alcohol Consumers M (SD)	Alcohol Abstainers M (SD)
**Resilience**		
Mental resilience	3.38 (0.88)	3.29 (0.93)
**Mood**		
Depression	9.49 (10.60)	9.13 (11.06)
Anxiety	5.80 (7.46)	6.49 (8.50)
Stress	10.65 (9.83)	10.68 (9.90)
**Coping**		
Problem-focused coping	2.25 (0.71)	2.36 (0.71)
Emotion-focused coping	2.04 (0.50)	2.06 (0.51)
Avoidant coping	1.61 (0.48)	1.53 (0.42)
**Personality**		
Extraversion	3.07 (1.07) *	2.78 (1.07) *
Agreeableness	3.55 (0.89)	3.59 (0.86)
Conscientiousness	3.76 (0.87)	3.82 (0.97)
Neuroticism	2.88 (1.11)	2.89 (1.16)
Openness to experiences	3.59 (0.86)	3.51 (0.90)

* *p* < 0.01 between alcohol consumers and alcohol abstainers.

**Table 3:** Means and standard deviations of individual hangover items and overall hangover severity.

Hangover Severity	M (SD)
Fatigue	6.3 (2.3)
Thirst	6.0 (3.0)
Concentration problems	4.6 (2.9)
Apathy	4.3 (3.0)
Nausea	3.9 (3.0)
Clumsiness	3.8 (3.1)
Sweating	3.4 (3.1)
Dizziness	3.3 (3.1)
Confusion	3.1 (2.8)
Stomach pain	3.0 (3.2)
Heart pounding	2.7 (2.9)
Shivering	1.3 (1.9)
AHSS total score	3.8 (2.2)

**Table 4:** Psychological constructs and their association with hangover severity.

Psychological Constructs	Hangover Severity	
	r	*p*-Value
**Resilience**		
Mental resilience	−0.305	0.004 *
**Mood**		
Depression	0.538	*p* < 0.001 *
Anxiety	0.602	*p* < 0.001 *
Stress	0.536	*p* < 0.001 *
**Coping**		
Problem-focused coping	0.149	0.183
Emotion-focused coping	0.278	0.013
Avoidant coping	0.386	*p* < 0.001 *
**Personality**		
Extraversion	−0.051	0.644
Agreeableness	−0.015	0.892
Conscientiousness	−0.234	0.031
Neuroticism	0.213	0.052
Openness to experiences	0.003	0.980

Significance (*p* < 0.0042 after Bonferroni’s correction) is indicated by *.

## Data Availability

The data presented in this study are available on request from the corresponding author.
